# Genetic Basis of Sudden Unexpected Death in Epilepsy

**DOI:** 10.3389/fneur.2017.00348

**Published:** 2017-07-20

**Authors:** Richard D. Bagnall, Douglas E. Crompton, Christopher Semsarian

**Affiliations:** ^1^Agnes Ginges Centre for Molecular Cardiology, Centenary Institute, Sydney, NSW, Australia; ^2^Sydney Medical School, University of Sydney, Sydney, NSW, Australia; ^3^Department of Neurology, Northern Health, Melbourne, VIC, Australia; ^4^Epilepsy Research Centre, Department of Medicine, University of Melbourne, Austin Health, Melbourne, VIC, Australia; ^5^Department of Cardiology, Royal Prince Alfred Hospital, Sydney, NSW, Australia

**Keywords:** sudden unexpected death in epilepsy, genetics, sudden death, cardiovascular disease, tonic–clonic

## Abstract

People with epilepsy are at heightened risk of sudden death compared to the general population. The leading cause of epilepsy-related premature mortality is sudden unexpected death in epilepsy (SUDEP). Postmortem investigation of people with SUDEP, including histological and toxicological analysis, does not reveal a cause of death, and the mechanisms of SUDEP remain largely unresolved. In this review we present the possible mechanisms underlying SUDEP, including respiratory dysfunction, cardiac arrhythmia and postictal generalized electroencephlogram suppression. Emerging studies in humans and animal models suggest there may be an underlying genetic basis to SUDEP in some cases. We will highlight a mounting body of evidence for the involvement of genetic risk factors in SUDEP, with a particular focus on the role of cardiac arrhythmia genes in SUDEP.

## Introduction

People with epilepsy have a twofold to threefold increased risk of premature mortality compared to the general population, which is attributed to factors both related and unrelated to epilepsy (1, 2). The most common cause of death that is related to epilepsy is sudden unexpected death in epilepsy (SUDEP), defined as “a sudden, unexpected, witnessed or unwitnessed, non-traumatic, and non-drowning death in patients with epilepsy with or without evidence for a seizure, and excluding documented status epilepticus, in which postmortem examination does not reveal a structural or toxicologic cause of death” (3). The definition “probable SUDEP” applies when a postmortem is not performed, and “possible SUDEP” for people with limited information regarding the cause of death or with a competing cause of death. Cases in which cardiorespiratory arrest is reversed by resuscitation with subsequent survival for more than 1 h are termed “near SUDEP” (3).

Estimates in studies of the incidence of SUDEP vary widely owing to differences in the age range and epilepsy populations studied. In general populations of people with epilepsy, approximately 1 SUDEP occurs annually per 1,000 people, whereas the incidence is lower in studies limited to children with epilepsy. In hospital or clinic-based studies, up to 7 SUDEP cases occur annually per 1,000, reflecting the higher proportion of more severe and treatment refractory cases (2–5). Because the incidence of SUDEP is common in young adulthood, the public health burden in terms of years of potential life lost ranks second only to stroke among neurological conditions in the United States (6).

Case–control studies analyzing clinical variables associated with SUDEP have highlighted generalized tonic–clonic seizures as the major risk factor. In addition, a long history of epilepsy, young age at diagnosis, early adulthood (aged 20–40 years), intellectual disability, and male gender, are also associated with elevated risk (4–7). While awareness of the health burden and risk factors of SUDEP is increasing among patients, doctors, and the community, the underlying causes of SUDEP are unknown.

Because many SUDEP cases are unwitnessed, the exact sequence of events is not known for the majority of cases. However, when witnessed, SUDEP almost always occurs in the aftermath of a generalized tonic–clonic seizure, and people with genetic epilepsies associated with this seizures type may be at heightened SUDEP risk. During the seizure, depressed autonomic control of respiratory drive may result in severe oxygen desaturation and a rise in blood carbon dioxide levels. The adverse effects of cessation of breathing may be exacerbated by airway obstruction in people sleeping face down, as is a common circumstance in SUDEP. Furthermore, depressed activity of serotonin neurons may blunt the natural arousal response to increased blood carbon dioxide levels. A seizure may also have various effects on the cardiac rhythm, including shortening or prolongation of the QT interval and changes in the QT interval are associated with sudden death in the general population. SUDEP cases with pathogenic variants in genes that cause the long QT syndrome (LQTS) may have a cardiac arrhythmogenic basis. Profound postictal suppression of central nervous system function may leave the patient immobile and unable to respond to hypoxia and hypercapnea, with a failure to auto-resuscitate. Thus, SUDEP likely results from an unfortunate coincidence of precipitating factors that makes one seizure a terminal event following a lifetime of seizures with little or no impact on life.

## Mechanisms of Sudep

**Figure 1:**
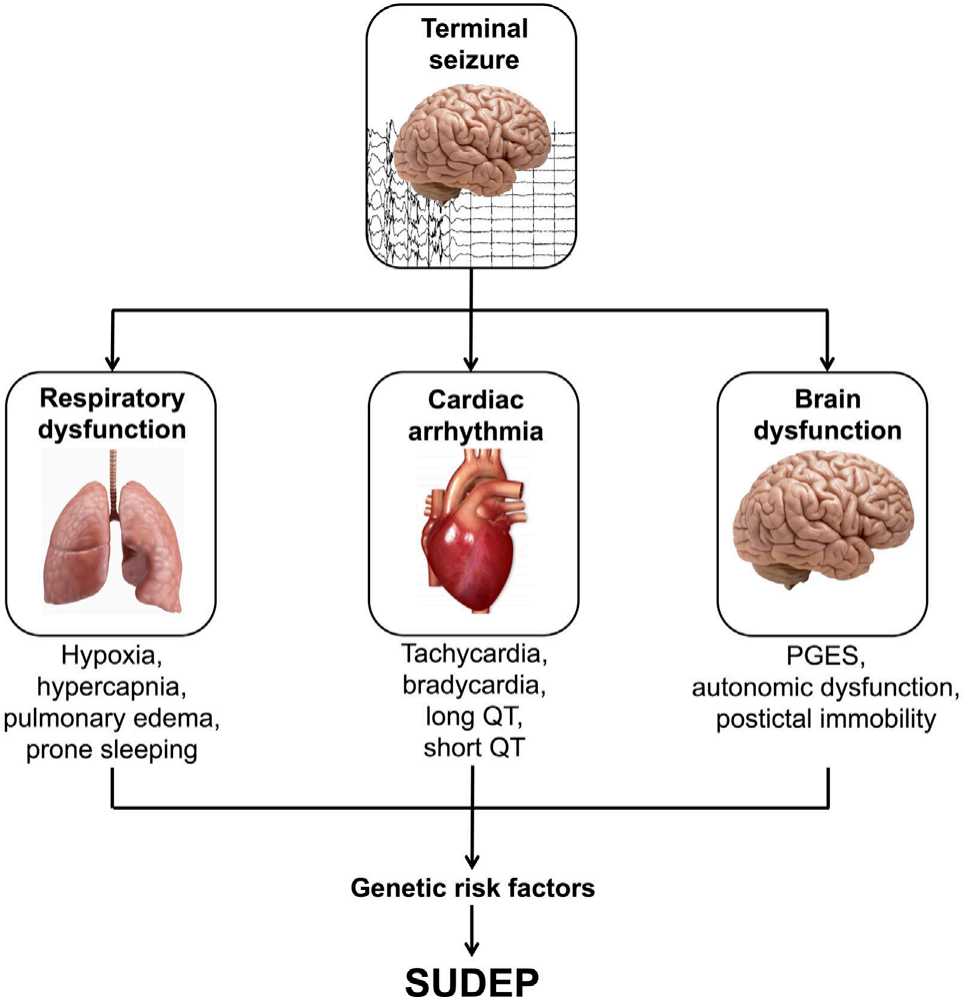
Possible mechanisms of sudden unexpected death in epilepsy (SUDEP). PGES, postictal generalized EEG suppression.

Recent data obtained from human studies and animal models have implicated severe alterations to respiratory, cardiac, and brain function as three possible mechanistic areas in SUDEP (Figure 1) (8–10).

### Respiratory Dysfunction

Inpatient video electroencephalogram (EEG) recordings of SUDEP occurring in epilepsy monitoring units show a fairly consistent sequence of respiratory and cardiac events preceding death. In the landmark MORTEMUS study of 16 SUDEP and 9 near SUDEP cases, generalized tonic–clonic seizures preceded an immediate and short phase of rapid breathing, then marked reduction in respiration rate with bradycardia and EEG suppression, followed by terminal apnea and asystole (4). In two-thirds of cases, there was a brief restoration of cardiac function, followed by progressive deterioration of respiration, apnea and asystole. Rarely, SUDEP may also occur without preceding epileptic seizures and follows a similar sequence of cardiorespiratory collapse as that seen in seizure-associated SUDEP (11). While the causes of respiratory dysfunction in SUDEP remain unresolved, physiological recordings of oxygen saturation and end-tidal carbon dioxide levels in people living with epilepsy have shown that generalized tonic–clonic seizures, which are the predominant terminal seizure type preceding SUDEP, can lead to transient respiratory arrest and apnea (12). One-third of seizures in 56 people with intractable localization-related epilepsy were associated with oxygen desaturation below 90%, and 11 seizures had severe oxygen desaturation below 70%, whereas the interictal baseline of oxygen desaturation in these people never fell below 90% (12). Furthermore, the extent of oxygen desaturation was correlated with seizure duration and was accompanied by an increase in end-tidal carbon dioxide levels in people with available data (12).

Centrally mediated apnea is more common than obstructive apnea during seizures. The forebrain sites underlying seizure-evoked hypoventilation were explored in three people with intractable epilepsy undergoing intracranial electrode and respiratory monitoring (13). When seizure activity spread to the amygdala, or on electrical stimulation of the amygdala, the respiratory rate immediately declined from a baseline of 17 down to 1 breath per minute, and was accompanied by oxygen desaturation (13, 14). The people were awake and vigilant, but unaware of the collapse of respiratory rate, even when the duration of amygdala stimulation was increased to 47 s. Collectively, these studies suggest that seizures can lead to centrally mediated hypoventilation, causing hypoxemia and hypercapnia, and that seizure-related respiratory compromise is a key early event in SUDEP.

Prone sleeping and pulmonary edema are common findings in SUDEP and may further exacerbate the effects of seizure-induced hypoventilation. SUDEP occurs most often at night with people lying in bed in the prone position (4, 5, 15, 16), and people with a history of nocturnal seizures have a heightened risk of SUDEP (17). The majority of “near SUDEP” events of the MORTEMUS study occurred during the day, with cardiopulmonary resuscitation occurring within 3 min of cardiorespiratory arrest, whereas no SUDEP cases received such intervention within 10 min of apnea (4). Nocturnal supervision is a proposed protective factor in SUDEP (18), and this raises the possibility that SUDEP occurs more often at night simply because they are less promptly attended, and tending to these people may be sufficient to restore cardiorespiratory function. Pulmonary edema and congestion of the lungs is found in the majority of SUDEP (19–21). Chest X-ray examination of 11 people following a generalized tonic–clonic seizure showed postictal pulmonary edema in 7, and the extent of edema was associated with seizure duration (22). However, although the degree of pulmonary edema is considered to be mild in SUDEP, it may be a further compounding factor on the adverse effects of seizure-related respiratory insufficiency and prone sleeping.

### Cardiac Arrhythmia

Epilepsy and seizures can have adverse affects on cardiac function, which may play an important role in the pathophysiology of SUDEP. Seizures can induce cardiac changes, such as tachycardia, bradycardia, and prolongation of the QT interval, possibly due to seizure-related effects on the autonomic nervous system. Ictal tachycardia, generally defined as >100 beats per minute, preceding, during, or following the onset of seizures, is common, occurring in an average of 82% of patients studied (23). Ictal tachycardia leading to ventricular fibrillation has been documented in two “near SUDEP,” both of which required defibrillation (4, 24). Slowing of the heart rate during seizures, or ictal bradycardia, is much less common than ictal tachycardia and has mostly been observed in people with temporal lobe epilepsy (25, 26). Postictal bradycardia followed by apnea was a consistent finding among monitored SUDEP of the MORTEMUS study. Why some seizures lead to SUDEP is unresolved, but it is apparent that heart rate changes may vary from seizure to seizure in some people, whereas others may have a consistent pattern.

The QT interval is a measure of the duration of ventricular depolarization and repolarization, and a prolonged or shortened QT interval is associated with sudden death risk in the general population (27, 28). The QT interval may become prolonged or shortened in people with refractory epilepsy (29), following generalized tonic–clonic seizures (30), or in association with seizure-associated oxygen desaturation (31), and seizure-related QT changes have been proposed to be involved in SUDEP (32, 33). However, in one retrospective study of 19 people with epilepsy who later died of SUDEP, the proportion of people with seizure-related prolongation of the QT interval was similar to those who had not died (34).

Long QT syndrome is an autosomal dominant disorder which affects 1 in 3,000 of the general population and is characterized by prolongation of the corrected QT interval on the electrocardiogram (35). Approximately 75% of LQTS is caused by pathogenic variants in the potassium ion channel subunits *KCNQ1* (LQT1, 35%) and *KCNH2* (LQT2, 30%), and the sodium ion channel subunit *SCN5A* (LQT3, 10%). Importantly, familial LQTS can lead to syncope, seizures, and in the most severe cases, sudden cardiac death (36). When LQTS is compared to SUDEP, there exist some parallels in the circumstances of death (Table 1). Of note, both in SUDEP and familial LQTS, the sudden death event is unexpected, frequently occurs at rest, or in bed, and the postmortem findings are identical in that no cause is identified and the heart appears structurally and histologically normal. Emerging evidence suggests that there may be an association between LQTS and epilepsy (37–40). Key causal genes of LQTS encode ion-channels that are expressed in the heart and the brain. *KCNQ1* transcripts are found in the adult human brain, and kcnq1 protein in the mouse is found in pyramidal neurons in CA1 to CA3, granule cells of the dentate gyrus, and hilar interneurons (41). Full-length *KCNH2* transcripts, and a primate-specific brain isoform, are found in human hippocampus, and *KCNH2* protein expression was confirmed in human hippocampus and frontal cortex using western analysis (42). *SCN5A* transcripts are found in human brain, and scn5a protein expression in the rat is detected in the ventral medial, dorsal medial, and posterior hypothalamic nuclei (43). Seizure episodes are common in LQTS, and particularly in LQTS type 2; however, while seizures in LQTS may be related to arrhythmia-mediated cerebral hypoxia, 1.6% of people with LQTS have EEG-documented seizure activity (37). As discussed below, a subset of SUDEP cases have rare variants in common genes responsible for LQTS and mice with a mutation in the LQTS type 1 gene, *kcnq1*, have seizures and sudden death (41).

**Table 1:** Comparison of sudden unexpected death in epilepsy (SUDEP) with familial long QT syndrome (LQTS).

Clinical feature	SUDEP	LQTS
Male predominance	Yes	Yes
Heart at postmortem	Normal	Normal
QT interval changes on ECG	Yes	Yes
Circumstances of death	Often unwitnessed, in bed	LQTS3 often at rest
Cause of death at postmortem	Unascertained	Unascertained

*LQTS3—long QT syndrome type 3 caused by SCN5A mutations*.

### EEG Suppression

Recordings from monitored SUDEP have shown that the EEG often shows global, persistent attenuation after seizures end, i.e., postictal generalized EEG suppression, suggesting that cerebral compromise could be a key mechanism of SUDEP. Generalized tonic–clonic seizures are the major risk factor for SUDEP across case–control studies and are associated with postictal generalized EEG suppression (44). In a case–control study of 10 SUDEP and 30 people with epilepsy, the duration of postictal generalized EEG suppression was longer in the seizures of SUDEP cases, and for each 1-s increase in duration, the odds of SUDEP increased by a factor of 1.7% (45). It was postulated that the profound and prolonged electrical shutdown of the brain might result in a tendency to central apnea. However, in another study of 17 presurgical patients who died with SUDEP and matched alive control patients, postictal generalized EEG suppression was not an independent risk factor for SUDEP (44). Furthermore, while postictal generalized EEG suppression was linked to the duration and extent of oxygen desaturation, there was no link with the coincidence of apnea (46). This has lead to an alternative proposal, in which postictal generalized EEG suppression is related to the severity of seizure-related intrinsic pulmonary dysfunction, rather than central apnea. This notion was supported in a study of 70 people with generalized tonic–clonic seizures in whom postictal generalized EEG suppression and postictal immobility were associated with respiratory dysfunction, and in which there was only a short duration of ictal apnea (47).

Most people who fit the high-risk profile of SUDEP do not die, and it is not clear why some seizures lead to SUDEP while others do not. Case reports and case–control studies of SUDEP have enhanced our understanding of SUDEP and its risk factors. However, inconsistencies between clinical associations likely relate to confounding factors such as the ascertainment of highly selected clinical or presurgical groups and the different seizure types, anti-epileptic medications, and study methodologies. Furthermore, the number of cases studied tends to be limited by the paucity of SUDEP cases with available phenotype data. Most likely, cardiorespiratory rates vary between seizures and patients; whether a seizure leads to SUDEP probably involves an unfortunate and interrelated combination of mechanisms, with additional environmental and genetic risk factors.

## Evidence for Genetic Risk Factors in Sudep

While SUDEP does not show a familial tendency, with two possible exceptions (48, 49), genetic analysis of SUDEP cases has been performed in a small number of studies to search for possible genetic risk factors, with a number of genes implicated (Table 2). With the availability of low cost, high throughput DNA sequencing, studies have progressed from single “candidate gene” -based studies (16, 50), to sequencing all 22,000 protein-coding genes, i.e., the exome (51), or the whole genome (52). A limiting factor is the availability of sufficient DNA from SUDEP cases, which is either collected during life of the patient or extracted from postmortem blood. There is increasing awareness of the importance of collecting a blood sample in the setting of unexplained sudden cardiac death, as postmortem genetic testing of cardiac arrhythmia genes, i.e., the molecular autopsy, can identify additional causes of sudden cardiac death over autopsy alone (53, 54). Most recently, DNA extracted from formalin-fixed, paraffin-embedded postmortem tissue that was stored for up to 14 years has been successfully exome sequenced (55). This study included the fixed postmortem tissue of one SUDEP in a person with Dravet syndrome in which the molecular autopsy identified a pathogenic *SCN1A* variant. This raises the exciting prospect that historical collections of postmortem fixed tissue may hold untapped pools of SUDEP cases suitable for genetic analysis and for which phenotyping data are available. Furthermore, this study offers encouragement that postmortem fixed tissue may provide an alternative source of DNA for exome sequencing-based molecular autopsy when postmortem blood or high quality extracted DNA is not available. Nevertheless, we recommend that postmortem blood should be kept in all SUDEP cases as a source of high quality DNA for genetic analysis.

**Table 2:** Genes associated with sudden unexpected death in epilepsy (SUDEP).

Gene	OMIM disease	Evidence for association with SUDEP
*KCNA1*	Episodic ataxia/myokymia syndrome	Animal model; variant found in SUDEP case
*SCN1A*	Dravet syndrome	Animal model; *de novo* variants found in SUDEP cases
*SCN2A*	Early-infantile epileptic encephalopathy 11	*De novo* variants found in SUDEP cases
*SCN8A*	Early-infantile epileptic encephalopathy 13	Animal model; *de novo* variants found in SUDEP cases
*DEPDC5*	Familial focal epilepsy with variable foci	*De novo* variants found in SUDEP cases
*KCNQ1*	Long QT syndrome type 1	Variants found in SUDEP cases
*KCNH2*	Long QT syndrome type 2	Variants found in SUDEP cases
*SCN5A*	Long QT syndrome type 3	*De novo* variant found in SUDEP case

### Inherited Cardiac Arrhythmia Genes

The first support for involvement of LQTS gene variants in SUDEP was on finding a novel *SCN5A* Arg523Cys variant following sequencing of five LQTS-associated genes in four SUDEP cases (56). The female patient had experienced generalized tonic–clonic seizures from age 17 years, involving bilateral synchronous epileptogenic activity on EEG; she was found dead in bed at age 25 years. Variants in *SCN5A*, encoding sodium voltage-gated channel alpha subunit 5, cause Brugada syndrome and LQTS type 3, which is associated with arrhythmias during rest or sleep (57).

Further support for LQTS variants in SUDEP was provided with a larger retrospective review of postmortem reports at a single forensic center between 1993 and 2009, which identified 22 SUDEP and 46 possible SUDEP cases, with DNA available on 48 cases (16). Sequencing of the three common LQTS genes, *KCNQ1, KCNH2*, and *SCN5A*, identified two rare non-synonymous variants. These were a *KCNH2* Arg176Trp variant, which has been reported as a founder mutation associated with a prolonged QT interval in Finnish LQTS families, and alters ion-channel activity *in vitro*, and an *SCN5A* Pro1090Leu, which has been reported previously in LQTS and in a case of sudden cardiac death (16). Further analysis of the same cohort was performed with sequencing of the family of four hyperpolarization-activated cyclic nucleotide-gated cation channel genes (*HCN1-4*) (50). HCN channels are voltage-gated ion channels primarily involved in the generation of spontaneous rhythmic activity in both cardiac pacemaker and neuronal cells, and genetic variants in *HCN1, HCN2*, and *HCN4* genes have been reported to account for familial sinus bradycardia and familial epilepsy syndromes. Three rare non-synonymous variants were identified; Phe738Cys and Pro802Ser in *HCN2* and Gly973Arg in *HCN4*, all of which were located in the cytoplasmic tail region of the proteins (50). Since the ascertained cases were de-identified, there was no opportunity to contact the surviving family to perform clinical phenotyping or co-segregation studies. The finite source of DNA and Sanger sequencing approach limited the analysis of additional genes in these people with SUDEP.

**Figure 2:**
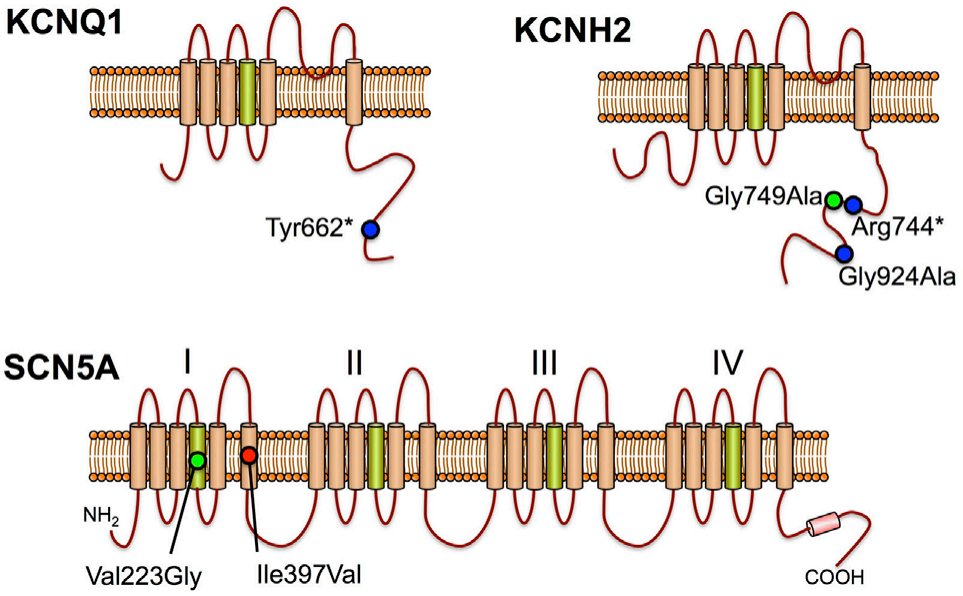
Long QT syndrome pathogenic variants in sudden unexpected death in epilepsy cases KCNQ1, KCNH2, and SCN5A proteins are shown with positively charged transmembrane segments in green. Positions of previously reported pathogenic mutations (blue circles), *de novo* missense mutation (red circle), and candidate pathogenic variants (green circles) are shown.

The largest SUDEP cohort with genetic investigation involved exome sequencing-based analysis of cardiac arrhythmia genes and epilepsy genes in 61 SUDEP cases (51). Importantly, the majority (*n* = 54) were classified “definite SUDEP” and 27 were previous participants of the Epilepsy Research Program, Melbourne, with detailed epilepsy phenotyping data and access to parental DNA for six. Of the remaining SUDEP, 15 were prospectively collected and 19 retrospectively collected coronial cases. The cohort comprised 56% males and the mean age at SUDEP was 28 years, with 27 out of 28 people found dead in bed in the prone position for which this information was available. There was a range of epilepsies phenotyped during life from the Melbourne cohort, but none had a diagnosis of cardiac disease. Analysis of exome sequencing data focused on the 3 common LQTS genes, 29 additional cardiac arrhythmia genes, 5 genes involved in central control of ventilation, and 72 epilepsy genes. Of particular note in cardiac arrhythmia genes, four pathogenic and two candidate pathogenic variants were found in the three key LQTS genes, including a *de novo SCN5A* Ile397Val variant, a Gly924Ala and Arg744* nonsense variant in *KCNH2*, and a Tyr662* nonsense variant in *KCNQ1* (Figure 2). The *KCNQ1* and *KCNH2* variants had been previously reported in people with LQTS and were absent in over 60,000 population controls, whereas the *de novo SCN5A* variant occurred in a highly conserved transmembrane domain. These four variants were regarded as highly likely to be pathogenic for LQTS and were found in one coronial SUDEP case and three SUDEP from the Melbourne Epilepsy Research Centre cohort.

In a further analysis of the exome data, the number of rare variants in each one of 22,000 genes from 58 European SUDEP cases and 2,936 European people who did not have epilepsy were compared, to test for genes enriched with rare variants. Although no gene reached genome-wide significance after correcting for multiple testing, the LQTS type 2 gene, *KCNH2* (*p* = 0.0037), was among the top 30 genes with the greatest number of rare variants in SUDEP compared to controls. People with LQTS type 2 are more likely to have a personal diagnosis of epilepsy history and a seizure phenotype compared to other types of long QT (37, 38), and this may account for the higher number of *KCNH2* variants in this cohort of SUDEP.

The finding of LQTS variants in SUDEP raises a number of questions: do variants in LQTS genes expressed in the brain and heart cause epilepsy as well as arrhythmias and sudden cardiac death? Is the sequence of cardiorespiratory events preceding SUDEP in people with epilepsy and LQTS variants different from those without LQTS variants? Does finding a LQTS variant in a person with epilepsy and sudden unexpected death following a molecular autopsy mean that the death should be reclassified as a sudden cardiac death? The finding of LQTS variants in SUDEP also has important implications for the surviving family members, both with and without epilepsy, who risk inheriting the variant and, therefore, being at increased risk of arrhythmias and sudden death. If an LQTS variant is found in a person with epilepsy during life there may be important clinical implications, including avoidance of medications that may prolong the QT interval, selection of antiarrhythmic drugs such as beta-blockers, and interventions for potentially lethal cardiac arrhythmias, such as implantable cardioverter defibrillator therapy to prevent sudden death. Importantly, SUDEP in people with epilepsy and LQTS variants may be predictable and preventable.

### Genetic Epilepsies with Increased SUDEP Risk

Some genetic epilepsies have a high incidence of SUDEP and the associated pathogenic variants may represent convenient biomarkers of SUDEP risk. In Dravet syndrome, >80% of people have a variant in *SCN1A*, encoding a neuronal voltage-gated sodium channel alpha subunit 1, with 95% of 80 tested variants arising *de novo* (58). There is a high mortality rate and SUDEP is the most common cause of death, with 59% of deaths due to SUDEP in a cohort of 100 Dravet syndrome who were followed for 1,073 person-years (59). This constitutes a Dravet-specific SUDEP rate of 9.32 per 1,000 person-years, which is among the highest SUDEP incidence rate reported in selected groups. Risk factors of SUDEP are common features of Dravet syndrome, including generalized tonic–clonic seizures, early seizure onset, and polytherapy. Furthermore, heart rate variability, a measure of sino-atrial node activity, was decreased in two cohorts of 15 and 20 Dravet syndrome compared to people with other epilepsies and healthy people, and this may increase susceptibility to cardiac conduction disease (60, 61). Mice heterozygous for a partial deletion of *scn1a*, or an *scn1a* nonsense variant, recapitulate the features of Dravet syndrome, including sudden death, and have been proposed as models of SUDEP (62, 63). In both of these mouse models, the circumstances of spontaneous or provoked seizures preceding death are similar to SUDEP in humans, in that generalized tonic–clonic seizures are followed by marked bradycardia and death. Mice that died had a higher number of seizures in the preceding 24 h compared to mice that did not die. Mice with global deletion of *scn1a*, or selective deletion of *scn1a* from the forebrain, have reduced heart rate variability, as in human Dravet syndrome (61), whereas *scn1a* deletion from the ventricles causes increased heart rate variability.

Variants in the brain and cardiac expressed voltage-gated sodium channel alpha 8 gene, *SCN8A*, cause early-infantile encephalopathy, with seizure onset between birth and 12 months of age (64). SUDEP occurs in 10% of reported cases, and SUDEP risk factors, such as generalized tonic–clonic seizures and intellectual disability, were present in most of these people (64, 65). Close to 1% of epileptic encephalopathy have *SCN8A* missense variants, typically located in the highly conserved transmembrane segments, and more than 60 have arisen as *de novo* mutations (65). Functional studies suggest that *SCN8A* missense mutations may cause impaired channel inactivation and persistent sodium current, which may increase neuronal excitability and firing (64). A *de novo SCN8A* Asn1768Asp variant found in a child with ataxia, intellectual disability, early-onset epileptic encephalopathy and SUDEP, was incorporated into a mouse model (52). Mice heterozygous for this variant show ataxia, as found in the child with this variant, and up to three seizures per day from age 2–3 months, which progress to SUDEP within the following month (66). There was incomplete penetrance, with half of the heterozygous mice showing no seizures after 6 months, whereas all mice homozygous for the missense variant have SUDEP, consistent with a gene dosage effect. The mice have increased persistent sodium current and neuronal hyperexcitability, in keeping with functional studies of *SCN8A* missense variants in transfected cells. *SCN8A* is expressed in the heart, and myocytes from mice with the *SCN8A* Asn1768Asp variant showed a lowered threshold for action potential firing and an increased incidence of delayed after-depolarizations. Two of three mice that had monitored death showed severe bradycardia preceding asystole (67).

As previously mentioned, exome-wide variant burden analysis of 59 SUDEP and almost 3,000 people without epilepsy was performed to identify genes with an excess of rare variants (51). Epilepsy genes showing a high number of variants with this analysis included another of the neuronal voltage-gated sodium channel genes, *SCN2A*, whereas the familial focal epilepsy gene, *DEPDC5*, was ranked first when considering variants that appear only once in the entire dataset. Variants in *SCN2A* cause Ohtahara syndrome and unclassified early-onset epileptic encephalopathies (68). At least three SUDEP with variants in *SCN2A* have been reported, including two with *de novo* mutations (51, 69). It remains to be determined why the early onset epileptic syndromes caused by variants in voltage-gated sodium channels, *SCN1A, SCN2A*, and *SCN8A* should have a high risk of SUDEP, but may be directly related to their effects on cardiorespiratory function, or their association with known SUDEP risk factors, such as generalize tonic–clonic seizures, young age at seizure onset, and intractable epilepsy.

Six out of 61 SUDEP had a rare or novel variant in *DEPDC5*, including 4 with nonsense variants, which accounted for the higher number of *DEPDC5* variants in SUDEP compared to people without epilepsy. Further *DEPDC5* nonsense variants have been reported in two brothers with SUDEP (48) and in one individual from a large French family with focal epilepsy (70). *DEPDC5* encodes disheveled, Egl-10, and plekstrin domain-containing protein 5, a negative regulator of the mammalian target of rapamycin complex I (mTORC1), and nonsense mutations lead to increased mTORC1 activity.

These insights into possible genetic underpinnings of SUDEP highlight the value of the molecular autopsy of SUDEP and the possible value of genetic biomarkers of SUDEP risk. In particular, the finding of LQTS gene variants has important clinical implications for the surviving family members who may also have inherited the variant and also be at risk of sudden death. The high number of *de novo* variants in SUDEP suggests that sequencing analysis of family trios can reveal SUDEP high-risk alleles. We advocate postmortem genetic testing of SUDEP with analysis of previously implicated genes (Table 2) as an adjunct to the autopsy investigation.

### Respiratory Genes with SUDEP Risk

The link between genes associated with neuronal regulation of respiratory function and SUDEP stem primarily from studies of animal models. Serotonin and other neuropeptides modify excitability of the respiratory network, and excitation or inhibition of serotonin neurones can increase or reduce respiratory drive, respectively. Serotonin neurones are stimulated by increased carbon dioxide concentrations and dysfunction of serotonin neurons may impair the ventilatory response to hypercapnea, predisposing to SUDEP. Mice with a nonsense mutation in the serotonin receptor gene, *htr2c*, are susceptible to rare spontaneous and audiogenic tonic–clonic seizures, respiratory arrest and sudden death (71, 72). The DBA/2 mouse is also considered a useful model of SUDEP, since the mice exhibit convulsive audiogenic seizures followed by respiratory arrest and sudden death (73). DRBA/2 mice have reduced expression of specific serotonin receptors in the brain (74), and treatment with the serotonin reuptake inhibitor, fluoxetine, showed a dose-related reduction in the incidence of seizures and respiratory arrest (75).

In humans, seizure-related respiratory compromise during sleep, followed by bradyarrhythmia and apnea, is a common finding in monitored SUDEP. Congenital central hypoventilation syndrome is a potentially lethal autonomic nervous system disorder characterized by hypoventilation and impaired ventilatory response to hypercapnia and hypoxemia during sleep. An increased frequency of bradyarrhythmias has been reported in children with congenital central hypoventilation syndrome. Expansion of an alanine repeat in the homeobox gene *PHOX2B*, is the predominant cause of congenital central hypoventilation syndrome, with frameshift, nonsense, and missense mutations in *PHOX2B* accounting for a small proportion of cases. Subjects with smaller *PHOX2B* expansions can present in later life with nocturnal hypoventilation and some have coexistent epilepsy. The involvement of variants in *PHOX2B* in SUDEP is, therefore, an attractive hypothesis; however, no polyalanine repeat expansions or point mutations were identified in a large SUDEP cohort (76). Furthermore, no rare variants were identified in and additional five genes with plausible roles in central control of ventilation (*ASCL1, BDNF, EDN3, GDNF*, and *RET*) among 61 SUDEP cases (51).

## Conclusion

Sudden unexpected death in epilepsy is a tragic event and failure to identify a cause of death has major clinical, emotional, and psychological effects on the surviving family. The discovery, validation, and pathophysiological role of genetic variants in SUDEP is important for further understanding of risk factors and prediction in families of people with SUDEP. Exome or genome sequencing of SUDEP enables a search for variants in multiple genes related to epilepsy, cardiac arrhythmia, and respiratory function, with plausible roles in the underlying pathophysiology of SUDEP. Molecular autopsy has revealed a surprising number of SUDEP cases with variants in cardiac arrhythmia genes that may heighten risk of unexplained sudden death and SUDEP in people with LQTS mutations may be predictable and preventable. There are also a number of *de novo* mutations reported in cardiac arrhythmia and epilepsy genes, which has important implications for the surviving family members as the risk of sudden death will be low, and may partly explain why SUDEP is largely non-familial. The ultimate goal will be to use genetic screening of people with epilepsy to identify gene variants that could increase the risk of sudden unexpected death.

## Author Contributions

RB, DC, and CS wrote the manuscript and approved the final version.

## Conflict of Interest Statement

The authors declare that the research was conducted in the absence of any commercial or financial relationships that could be construed as a potential conflict of interest.
